# Novel APLNR antagonist candesartan induces tumor vascular normalization through ROS/cGAS/STING axis and augmented sunitinib response in breast cancer

**DOI:** 10.1186/s13046-025-03584-4

**Published:** 2025-11-28

**Authors:** Chenyu Liang, Mingzhu Wang, Tianxin Li, Yongtao Duan, Chuanjun Song, Yongfang Yao

**Affiliations:** 1 School of Pharmaceutical Sciences, Zhengzhou University, Zhengzhou, 450001 China; 2 Children’s Hospital Affiliated to Zhengzhou University, Zhengzhou University, Zhengzhou, 450018 China; 3 The First Dongguan Affiliated Hospital, Guangdong Medical University, Dongguan, 523710 China; 4 Henan Provincial Key Laboratory of Pediatric Hematology, Children’s Hospital Affiliated to Zhengzhou University, Zhengzhou University, Zhengzhou, 450018 China; 5 College of Chemistry, Zhengzhou University, Zhengzhou, 450001 China; 6 Pingyuan Laboratory, Zhengzhou, 450001 China; 7 Key Laboratory of Advanced Drug Preparation Technologies, Ministry of Education, School of Pharmaceutical Sciences, Zhengzhou University, Zhengzhou, 450001 China

**Keywords:** Tumor vascular normalization, APLNR, Drug repurposing, Antagonist, STING, Sunitinib

## Abstract

**Background:**

The tumor vasculature exhibits immature development characterized by inadequate pericyte coverage, resulting in excessive permeability, poor perfusion, and hypoxia. The aberrant vascular network not only facilitates metastasis but also confers therapeutic resistance. Accumulating evidence indicates that inducing tumor vascular normalization represents a promising antitumor strategy. Given the pivotal role of the APLN/APLNR axis in vascular maturation positions it as a promising therapeutic target for anti-tumor vascular therapy.This study aims to discover novel APLNR antagonists, investigate their effects on tumor vascular normalization, and evaluate their combinatorial efficacy with existing anticancer therapies.

**Methods:**

Initially, based on a drug repurposing strategy, we employed computer-aided virtual screening to identify compounds with high binding affinity to APLNR from the FDA-approved drug library. And further utilized a zebrafish retinal vascular abnormality model to screen out the candidate compound that could induce vascular normalization. Then, we assessed the impact of the candidate compound on vascular normalization by detecting the proliferation, migration and tubule formation abilities of HUVEC in vitro and investigating tumor vascular maturity, vascular perfusion and hypoxia in vivo. Additionally, we explored the molecular mechanisms underlying the regulation of vascular normalization by the candidate compound using western blot and comet assay. Finally, we employed a breast cancer mouse model to examine the antitumor efficacy of the candidate compound when used alone or in combination with Sunitinib.

**Results:**

Employing a drug repurposing strategy combined with computational screening, we identified Candesartan, an approved antihypertensive drug with a well-established safety profile, as a high-affinity APLNR antagonist. Additionally, in vitro, Candesartan significantly inhibited HUVEC proliferation, migration, and tube formation. In vivo, it promoted tumor vascular maturation, improved vascular perfusion, and alleviated hypoxia. Mechanistically, Candesartan activated the ROS/dsDNA/cGAS/STING pathway to drive vascular normalization. Notably, Candesartan synergistically potentiated sunitinib’s antitumor efficacy while inhibiting pulmonary metastasis and ameliorating Sunitinib-induced hepatotoxicity.

**Conclusion:**

This study provides a novel direction for APLNR antagonist development and demonstrates that combining Candesartan with the standard antiangiogenic agent Sunitinib leverages the advantage of drug repurposing, suggesting a promising and rapidly translatable strategy for optimized cancer therapy.

**Supplementary Information:**

The online version contains supplementary material available at 10.1186/s13046-025-03584-4.

## Introduction

The tumor vascular network is typified by vasodilation, tortuosity, and disorganization. Immature vascular development and lack of proper pericyte association result in excessive permeability, poor perfusion, and increased hypoxia [1]. These conditions upregulate hypoxia-inducible factor (HIF-1) and drive overexpression of pro-angiogenic genes like fibroblast growth factor (FGF) and vascular endothelial growth factor (VEGF), while significantly reduce the production of angiogenesis inhibitory factors [2, 3]. Consequently, the angiogenic process is skewed towards a pro-angiogenic state, fostering abnormal angiogenesis. Anti-angiogenesis therapy (AAT) was initially developed as monotherapy to starve tumors of nutrients [4, 5]. However, early clinical trials showed that bevacizumab, a VEGF antibody, failed to improve survival outcomes when used alone. In addition, the clinical use of Sunitinib can cause Treatment-related toxicities such as myelosuppression, hypertension and abnormal liver function [3, 6]. In contrast, its combination with other anti-tumor agents yielded improved treatment results. In 2001, Jain proposed the concept of “tumor blood vessel normalization“ [7].Since then, emerging evidences have suggested that the regulation of tumor vascular normalization may exert tumor-suppressive effects. For instance, DLL1 induces normalization of tumor blood vessels, characterized by sustained improvements in vascular perfusion and reduced hypoxia within the tumor microenvironment [8]. Similarly, VE-PTP inhibitors and simvastatin normalize tumor vasculature, delaying tumor growth and metastasis [9]. However, the number of tumor-targeting drugs focused on modulating vascular normalization remains limited, highlighting the need for new targets.

APLNR (APJ) is a G protein-coupled receptor, with endogenous ligands APLN (Apelin) and ELA (ELABELA). This receptor is widely expressed in cardiomyocytes, adipocytes, neuronal cells, and endothelial cells [10]. In myocardial cells, specifically, under hypoxic conditions, exogenous APLN has been shown to regulate autophagy, proliferation, and migration of pulmonary artery smooth muscle cells (PASMCs) via the PI3K/Akt/mTOR signaling pathway. These findings underscore the multifaceted significance of APLN in orchestrating vascular responses to hypoxia, and highlight its potential as a therapeutic target for ischemic cardiovascular diseases. In vascular endothelial cells, APLN/APLNR axis promotes endothelial cell proliferation and vascular maturation, exerting significant vascular protective effects [11, 12]. For instance, reduced plasma APLN levels are associated with essential hypertension and left ventricular dysfunction [13]. The downregulation of APLN expression in atherosclerotic lesions is associated with the development of coronary collateral circulation in patients with stable angina [14]. These findings further underscores its pivotal role in modulating vascular function. Thus, indicating that APLN/APLNR signaling pathway play a key role in maintaining cardiovascular homeostasis. Furthermore, Studies demonstrate that APLNR serves as an angiogenesis indicator in colorectal cancer and reduces tumor angiogenesis in lung and breast cancer [15].Which positioning APLNR as a potential target for anti-tumor vascular therapy.

Inhibitors targeting the APLN/APLNR axis can be categorized into three types, include antibody, peptide, and chemical small molecule inhibitors. Such as, ALX40-4 C, a synthetic polyarginine peptide stabilized by terminal protection and D-amino acids, exhibits low-affinity inhibition of APLNR binding, impairing receptor internalization and signal transduction [16]. Amodiaquine, a selective small molecule APLNR antagonist identified through high-throughput screening, inhibits APLN/APLNR signaling in a concentration-dependent manner and reduces pathological choroidal neovascularization in a mouse model [17]. Additionally, JN241, a single-domain antibody (sdAb) targeting APLNR, which constitutes the first reported co-crystal structure of a functional antibody bound to a class A G protein-coupled receptor (GPCR) [18]. As research progresses, APLNR inhibitors have shown remarkable efficacy in pathological neovascular diseases and have been validated as a novel target for anti-tumor therapeutics. However, the variety of available inhibitors is limited, and their role in tumor vascular normalization is not fully elucidated.

This study aimed to discover novel APLNR inhibitors, examine their role in tumor vascular normalization, and assess their combinatorial potential with existing anti-cancer therapies. Drug repurposing is an innovative strategy in drug development, which by uncovering the new therapeutic potential of already marketed drugs and reusing the existing clinical safety data, can significantly accelerate the clinical transformation process of drugs. Given that, we employed the drug repurposing approach to discover drug entities with the function of modulating tumor vascular normalization by antagonizing APLNR. Computational virtual screening of FDA-approved drug libraries identified candesartan as a candidate compound with high APLNR-binding affinity and potent pro-vascular normalization activity. The effect of candesartan on tumor vascular normalization was evaluated in vivo by using an orthotopic breast cancer model. Notably,

Candesartan combined with Sunitinib, not only enhanced anti-tumor efficacy but also significantly suppressed tumor pulmonary metastasis and alleviated Sunitinib-induced liver damage in mice. These findings provide insights for developing novel APLNR inhibitors and establish a theoretical foundation for combination therapy strategies utilizing APLNR inhibitors to potentiate anti-tumor effects through inducing tumor vascular normalization.

## Methods

### Virtual screening and molecular docking

Based on the determined APLNR protein structure (PDB ID: 7SUS), docking optimization was conducted. Utilizing the MOE software, a screening process was performed on G protein-coupled receptor drugs that have been approved by the FDA. Compounds predicted to have high binding affinity were designated as candidates for biological validation.

### Zebrafish model of hypoxia-induced retinal vascular defects and compound toxicity screening

Transgenic zebrafish Tg(flk: EGFP) were obtained from the China Zebrafish Resource Center and maintained at 28 ± 1 °C under a 14-hour light/10-hour dark cycle. To assess compound toxicity, embryos (20 per well in 24-well plates) were exposed from 12 to 48 h post fertilization (hpf) to a series of concentrations of test compounds in E3 medium, with the APLNR antagonist F13A serving as a positive control. Survival rates were recorded to calculate the median lethal concentration (LC₅₀). For the retinal angiogenesis assay, embryos at 1 day post fertilization (dpf) were transferred to E3 medium containing 5 mM CoCl₂·6 H₂O and 2.5 µg/mL GS4012 to induce hypoxia and abnormal vessel formation. At 3 dpf, test compounds were added and incubation continued until 5 dpf. Blank, model, and vehicle (≤ 0.1% DMSO) controls were included, with at least three replicates per group (*n* = 10–15 embryos). At 5 dpf, retinal vasculature was imaged using fluorescence microscopy and analyzed for branching number, vessel length, and density using ImageJ. Data are presented as mean ± standard deviation (SD) and analyzed using one-way ANOVA followed by Tukey’s post hoc test, with *p* < 0.05 considered statistically significant.

### Cell culture

HUVEC, 4T1 and HEK293T were acquired from the National Experimental Cell Resource Sharing Service Platform (Beijing). HUVEC, 4T1 and HEK293T are maintained in Dulbecco’s modified Eagle’s medium (DMEM) (KeyGEN, KGL1206-500) supplemented with 10% fetal bovine serum (KeyGEN, KGL1206-500,) and 1% penicillin and streptomycin. All cells were cultured in 37 °C humidified incubators with 5% CO_2_.

### cAMP assay

A suspension of APLNR-HEK293T cells was prepared and seeded into a 384-well OptiPlate™ 384 at a density of 1 × 10⁴ cells per well. The cells were cultured at 37℃ for 24 h. The culture medium was then removed, and 7.5 µL of serum-free medium containing 500 µM IBMX and the test compound was added to each well. The plate was incubated at 37℃ with 5% CO_2_ for 30 min. The concentration of cAMP was subsequently measured using the cAMP-Glo™ Assay Kit (Promega).

### FLIPR^®^ calcium 6 assay for APLNR antagonist screening

HEK293 cells stably expressing APLNR were seeded in 96-well plates (20,000 cells/well) and loaded with Calcium 6 dye (Molecular Devices) for 2 h. Candidate compounds dissolved in DMSO were applied at 2 µM for 15 min, followed by stimulation with the endogenous agonist Apelin-13 (100 nM). Real-time intracellular calcium flux was quantified using FLIPR Tetra^®^ System (470–495 nm excitation/515–575 nm emission).

### Surface plasmon resonance (SPR)

SPR, which is a reliable method for analyzing real-time biomolecular interactions without labeling, was utilized to identify the binding affinity between APLNR and Candesartan. Candesartan is diluted with sterile phosphate-buffered saline (PBS). The APLNR protein is immobilized on a CM5 sensor chip via coupling, and then different concentrations of Candesartan (12.5 µM, 6.25 µM, 3.125 µM, 1.5625 µM, 0.78125 µM, 0.390625 µM) are injected. The Biacore T200 system can continuously and real-time monitor the changes in resonance units (RU), thereby obtaining the binding situation of candesartan with APLNR. The entire sensorgram is fitted using a 1:1 kinetic binding model, and the equilibrium dissociation constant (KD) is calculated.

### Sulforhodamine B assay

The culture medium was aspirated from the plates, and cells were fixed with 10% trichloroacetic acid (TCA) for 30 min. The plates were then washed twice with distilled water and air-dried. Subsequently, 0.4% sulforhodamine B (SRB) solution was added, and the plates were stained on a shaker for 20 min. The staining solution was discarded, and excess dye was removed by washing with freshly prepared 1% acetic acid. After air-drying, 10 mM Tris solution was added to each well. The plates were agitated on a shaker for 5 min to dissolve the dye, and the absorbance was measured at 560 nm using a microplate reader.

### Wound healing assay

HUVEC cells were digested and inoculated into 6-well plates, with 1 × 10^5^ cells added to each hole. On the second day, the gun head was drawn vertically along the edge of the upper lid of the hole plate. The cells were washed twice with PBS, the scratched cells were removed, and the serum free medium containing drugs was added. The photos were taken and recorded under the microscope for 0 h and cultured in a 5% CO_2_ incubator at 37℃. After 48 h, the sample was taken and photographed for recording. The cell scratch area was calculated with Image J software.

### Transwell assay

Migration assays were performed using the Transwell cell culture insert. HUVEC (3 × 10^4^ cells) were suspended with 100µL serum-free medium containing different concentrations of compounds and placed in the upper layer of the Transwell cell culture insert, and 300µL medium containing 10% FBS was placed below the cell-permeable membrane. Following an incubation (48 h), the cells that had migrated through the membrane were stained with 0.2% crystal violet for 30 min and count.

### Western blotting analysis

Total protein was extracted using RIPA lysis buffer (Solarbio, China) supplemented with phenylmethylsulfonyl fluoride (PMSF; Thermo Fisher Scientific, USA), protease inhibitor cocktail (Roche, Switzerland), and phosphatase inhibitor cocktail (Roche, Switzerland). Protein concentration was determined using a BCA protein assay kit (Thermo Fisher Scientific, USA). Equal amounts of protein were separated by 10% SDS-polyacrylamide gel electrophoresis (SDS-PAGE) and transferred onto nitrocellulose (NC) membranes. After transfer, the membranes were blocked with 5% non-fat milk in TBST at 37 °C for 2 h, followed by overnight incubation at 4 °C with primary antibodies against FAK (catalog no. 71433 T), MMP-9 (catalog no. 13667 T), γ-H2AX (catalog no. 9718 T), STING (catalog no. 50494 T), TBK1 (catalog no. 3504 T), phospho-TBK1 (p-TBK1, catalog no. 5483 T), IRF3 (catalog no. 4302 T), phospho-IRF3 (p-IRF3, catalog no. 29047 T), and GAPDH (catalog no. 60004-1-Ig). All primary antibodies were purchased from Cell Signaling Technology (CST, USA), except for GAPDH, which was obtained from Proteintech (Wuhan, China). After washing, the membranes were incubated with HRP-conjugated goat anti-rabbit or anti-mouse IgG secondary antibodies (Biosharp, China) for 1 h at room temperature. Protein bands were visualized using an enhanced chemiluminescence (ECL) detection system and scanned using a chemiluminescence imaging system.

### Tube formation assay

The 96-well plate was coated with 50 µl matrix glue per well, and then HUVEC was inoculated into the 96-well plate coated with matrix glue with a density of 4 × 10^4^ cells per well. Photos were taken with an optical microscope (Nikon, China) after culture at 37℃ and 5% CO_2_ for 6–8 h, and the length of the generated blood vessels was analyzed using ImageJ.

### Alkaline comet assay

Logarithmic phase cells were plated in six-well plates, treated with drug-containing medium for 24 h, then collected and resuspended in PBS. Normal melting point agarose (NMA) and low melting point agarose (LMA) were prepared at 45℃ and 37℃, respectively. Frosted slides were preheated, coated with 100 µL NMA, and solidified at 4℃. Cell suspensions were mixed with LMA, layered on solidified NMA, and solidified again at 4℃. After lysis in chilled buffer overnight and washing with PBS, slides were subjected to alkaline unwinding and electrophoresis. Following neutralization and staining with EB, cell morphology was observed and imaged under a laser confocal microscope.

### ROS generation assay

Flow cytometry was performed to measure intracellular reactive oxygen species (ROS) levels using DCFH-DA (2’,7’-Dichlorodihydrofluorescein diacetate). Cells were collected and stained with DCFH-DA, followed by incubation at 37℃ for 30 min in the dark. Subsequently, the cell populations were analyzed by flow cytometry.

### Apoptosis assay

The Annexin V-FITC/PI Apoptosis Detection Kit (Vazyme) was used to detect cell apoptosis according to the manufacturer’s instructions. Cells were stained with the kit and then analyzed using flow cytometry (Becton Dickinson [BD]).

### Establishment of the breast cancer mouse model

Female BABL/c mice aged 6–8 weeks were purchased from Beijing SPF Biological Co., LTD. 4T1 cells in good growth condition were collected and injected in situ into the mammary fat pad of female BALB/c mice at a density of 2 × 10⁶ cells per mouse. When the tumor volume reached 100–300 mm^3^, the mice were randomly assigned into different treatment groups: control group (Vehicle), Candesartan treatment group (10,30 mg/kg), Sunitinib treatment group (40 mg/kg), and the combination treatment group. Candesartan was administered via intragastric gavage once daily for 14 consecutive days, while Sunitinib was administered via intragastric gavage once every two days for 14 days. When the tumor size increased to approximately 1200 mm^3^, we will conduct further tests. It should be pointed out that, when studying vascular leakage, DyLight™ 649-labeled tomato lectin (Tomoto-lectin) was injected intravenously into the mice via the tail vein two hours prior to tissue collection. The tumor tissues were then excised. Half of each tumor was fixed in 4% paraformaldehyde and subsequently processed for paraffin embedding and immunofluorescence staining. The remaining half was immediately frozen in liquid nitrogen and prepared as frozen sections for further experimental procedures. All animals were raised in an SPF environment. All animal testing is authorized by the Animal Ethics Committee of Zhengzhou University. We followed all the rules and regulations.

### Immunofluorescence

Paraffin-embedded mouse tumor tissue sections were deparaffinized, rehydrated, and fixed with 4% paraformaldehyde at room temperature for 20 min. After washing with PBS, the sections were permeabilized with 1% Triton X-100 (Solarbio, China) for 15 min at room temperature, followed by blocking with 5% bovine serum albumin (BSA) in PBS containing 0.1% Triton X-100 for 1 h at room temperature to reduce nonspecific binding. The sections were first incubated with primary antibodies against PECAM-1 (CD31) and α-smooth muscle actin (α-SMA), including Alexa Fluor 488-conjugated anti-CD31 antibody (clone SC-376764-AF488, Santa Cruz Biotechnology, Japan) and rabbit anti-α-SMA antibody (catalog no. AF0048, Beyotime Biotechnology, China). After washing, the sections were further incubated overnight at 4 °C with primary antibodies against dsDNA and HSP60. The next day, the sections were incubated with Alexa Fluor 647-conjugated goat anti-rabbit IgG (H + L) secondary antibody (catalog no. A0468, Beyotime Biotechnology, China) for 1 h at room temperature in the dark. Nuclear staining was performed using DAPI (catalog no. C0060, Solarbio, China). Finally, all slides were mounted with antifade mounting medium and observed using a STELLARIS 5 confocal microscope (Leica Microsystems, Germany).

#### Immunohistochemical

Immunohistochemical staining was performed on formalin-fixed, paraffin-embedded tissue sections. After deparaffinization and rehydration, antigen retrieval was conducted by heating the sections in citrate buffer (pH 6.0) at 95 °C for 12 min. Endogenous peroxidase activity was blocked by incubation with 3% hydrogen peroxide for 10 min at room temperature, followed by blocking of non-specific binding sites with 10% goat serum for 30 min at room temperature. The sections were then incubated overnight at 4 °C with a primary antibody against HIF-1α (rabbit anti-human, catalog no. 48085 T, Cell Signaling Technology, USA) diluted at 1:200. After washing, the sections were incubated with HRP-conjugated goat anti-rabbit IgG secondary antibody (catalog no. A0208, Beyotime Biotechnology, China) at a dilution of 1:500 for 30 min at room temperature. Color development was achieved using a DAB substrate kit, and the sections were counterstained with hematoxylin. After dehydration, clearing, and mounting with neutral resin, the stained sections were observed and imaged under an inverted microscope.

### Hematoxylin-Eosin staining

All tissues were soaked in 4% paraformaldehyde and fixed for 48 h. After gradient dehydration with ethanol, the tissues were then permeated with xylene and paraffin wax. Sample blocks were cut to 4 μm thick and stained with hematoxylin and eosin (H&E). Finally, the slide is observed under a bright field microscope.

### Picric acid staining for metastatic nodule detection

To observe metastasis, tissues were harvested and fixed in Bouin’s solution (saturated picric acid 75 ml + 40% formaldehyde 25 ml + glacial acetic acid 5 ml). After fixation, tissues were stained with picric acid to visualize metastatic nodules, which appeared as distinct lesions against the background tissue.

### Statistics

All experimental results were repeated three times, and statistical analysis was performed using GraphPad Prism 8.0 software. The significant difference between the two groups was tested by T test, and when there were more than three groups, the significance analysis was performed by One-way ANOVA. ^*^P < 0.05, ^**^P < 0.01, ^***^P < 0.001, ^#^*P* < 0.05, ^##^*P* < 0.01, or ^###^*P* < 0.001.

## Results

### Screening of compounds targeting APLNR to promote vascular normalization

A virtual screening of the FDA-approved drug library was conducted to identify potential APLNR inhibitors. Molecular docking simulations were performed on 2509 drugs against the resolved APLNR protein structure, with binding affinities scored to prioritize the top 21 candidate compounds (Table S1; DrugBank IDs provided). These candidates were further evaluated for APLNR inhibitory activity at the concentration of 2 µM by using a FLIPR^®^ Calcium 6 Assay Kit, which quantifies ligand-induced intracellular calcium flux. In this assay, APLNR activation by its endogenous agonist Apelin-13 triggers calcium mobilization, while test compounds competitively inhibit this response. Result showed that, 8 candidate compounds (A-2, A-3, A-4, A-6, A-7, A-9, A-11, and A-15) exhibited notable inhibition rates (from 48.1% to 72.3%), indicating their potential as potent APLNR inhibitors, and were further assessed for their effects on regulating vascular normalization in vivo.

The zebrafish (Danio rerio) model was employed due to its distinct advantages, the optical transparency, which enable in vivo vascular visualization. Notably, the zebrafish retinal vasculature exhibits significant similarities with human vascular architecture, making it a an ideal model for assessing compound-induced vascular normalization. Firstly, the 8 candidate compounds underwent preliminary toxicity evaluation in zebrafish embryos. Healthy zebrafish embryos were collected and allocated into 24-well plates at a density of 20 embryos per well. At 12 h post-fertilization (hpf), the culture medium was replaced with freshly prepared medium containing graded concentrations of each test compound. At 48 hpf, the number of surviving embryos was quantified, and the lethal dose 50 (LD_50_) for each compound was determined based on the survival data. F13A, a commercially available APLNR antagonist, served as the positive control. The resulting toxicity profiles are presented in Table1.

**Table 1:** Toxicity experiment on zebrafish

Compound	LD_50_(µM,48 h)	Compound	LD_50_(µM,48 h)
A-2	1.10 ± 0.12	A-3	42.10 ± 0.21
A-4	1.27 ± 0.35	A-6	2.38 ± 0.18
A-7	1.20 ± 0.01	A-9	10.2 ± 0.27
A-11	28.57 ± 0.16	A-15	0.49 ± 0.03
F13A	2.85 ± 0.31		

**Fig. 1:**
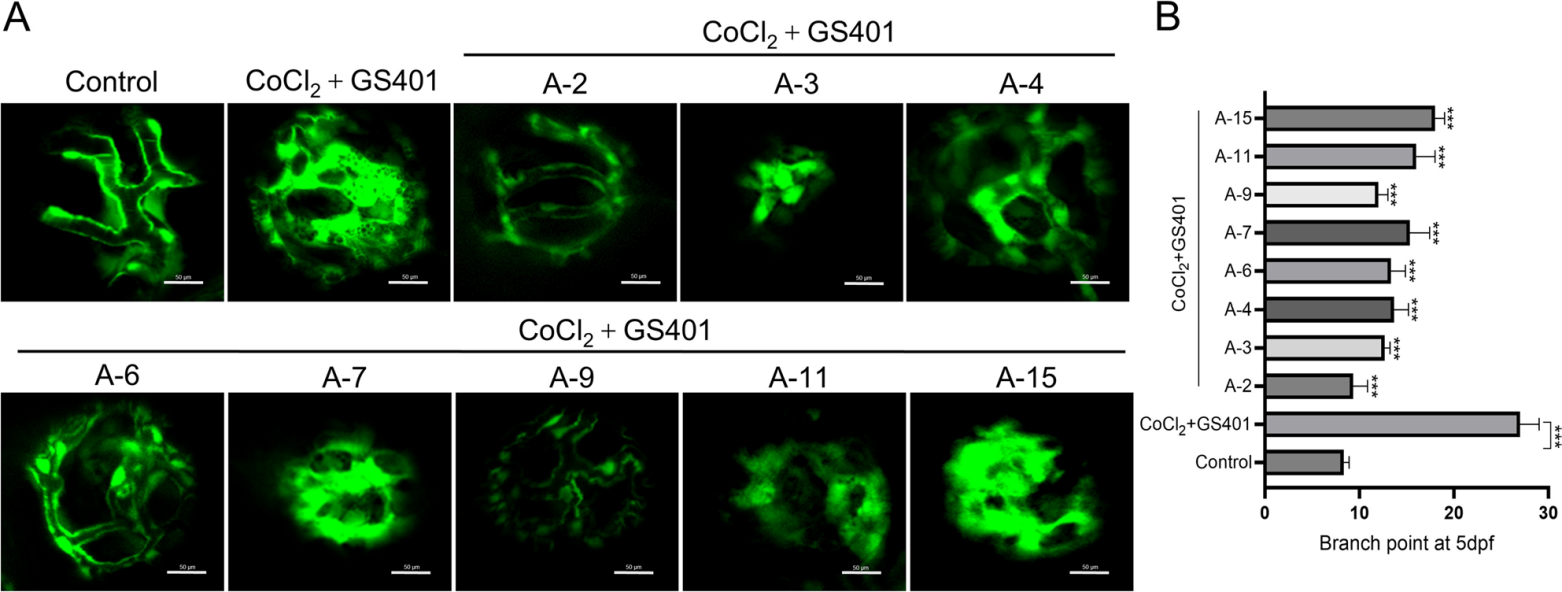
Candidate compounds were evaluated on zebrafish for their effects on vascular normalization. **A** Effects of candidate compounds on zebrafish vascular normalization, A-2 (1 µM), A-3 (40 µM), A-4 (1 µM), A-6 (2 µM), A-7 (1 µM), A-9 (10 µM), A-11 (25 µM), A-15 (0.4 µM). **B** Corresponding statistical histogram. Relative to CoCl_2_ + GS401 group, *n* = 3, ^***^ *P* < 0.001, Scale bars, 50 μm)

Furthermore, we established a hypoxia-induced vascular dysregulation model with CoCl_2_ (5mM) and a VEGF inductive agent GS401 (2.5 ug/ml) in zebrafish embryos to assess the capacity of candidate compounds to regulate vascular normalization. Following Zebrafish toxicity profiling, 8 candidate compounds were administered at optimized concentrations (< LD_50_) to treat the zebrafish vascular abnormality model separately. Retinal vasculature was visualized by confocal microscopy at 5 days post-fertilization (dpf), with subsequent morphometric analysis of vascular branching patterns and segment lengths. As demonstrated in Fig. 1, hypoxic conditions induced characteristic vascular pathology, manifesting as reduced vessel diameter, aberrant branching complexity, and loss of structural organization. While, 8 candidate compounds intervention markedly attenuated these pathological features, demonstrating significant reduction in ectopic branching and restoration of vascular architecture, especially compound A-2 (Fig. 1). Based on these quantitative assessments, compound A-2 (Candesartan, an angiotensin II receptor blocker, clinically used for the treatment of primary hypertension, with a well-documented safety profile in clinical use) emerged as the most promising candidate and was advanced for further investigation.

### Molecular docking and targeting validation of candesartan as an APLNR inhibitor

**Fig. 2:**
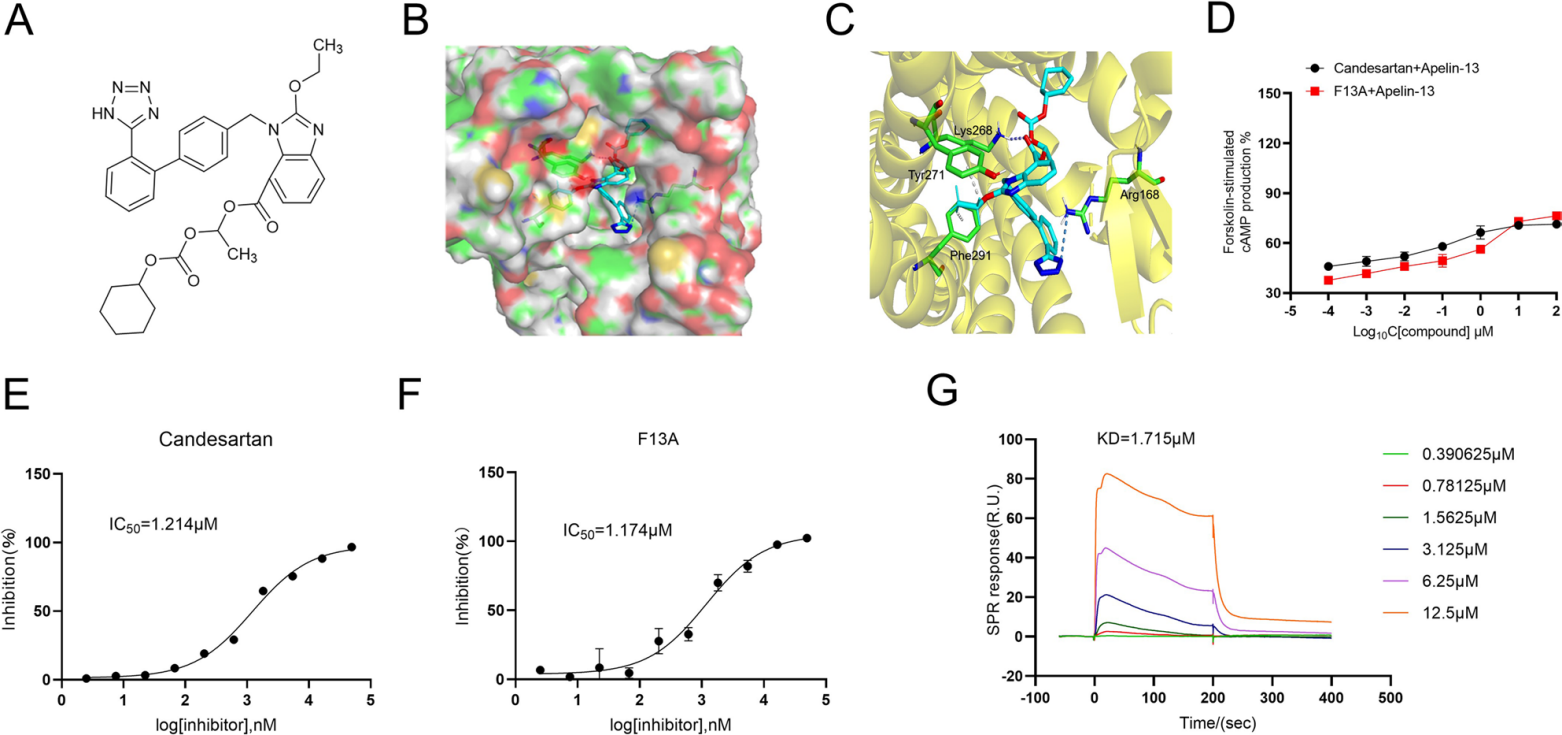
Binding modes of candesartan and APLNR. **A** The structural formula of candesartan; **B-C** Molecular docking and binding mode of candesartan with APLNR. **D **Protein validation results of APLNR-HEK293T overexpression cell line. **E-F** The inhibitory effects of F13A and Candesartan on the changes in intracellular calcium ion concentration induced by Apelin-13. **G** Affinity assay for candesartan and APLNR

To characterize the molecular interactions between candesartan and APLNR, we performed molecular docking simulations using the APLNR crystal structure (PDB: 7SUS) as a template. As illustrated in Fig. 2B and C, candesartan occupies the orthosteric binding pocket of APLNR, forming hydrogen bonds with Lys268 and Arg168, while establishing p-π interactions with Tyr271 and Phe291. These structural observations suggest high-affinity binding to the receptor’s active site. In addition, previous studies have established that APLNR activation by its endogenous agonist Apelin-13 inhibits Gi protein signaling, thereby attenuating forskolin-induced cAMP accumulation. Then, using APLNR overexpression cell line (HEK293T-APLNR) and cAMP quantification assays to investigate the inhibitory activity of candesartan on APLNR. We confirmed that stimulation of HEK293T-APLNR cells with forskolin (20 µM) significantly elevated cAMP levels, whereas Apelin-13 (0.1 µM) effectively reversed this effect, furthermore candesartan competitively inhibited Apelin-13-mediated cAMP mobilization. Results showed that, candesartan (10^− 4^−100 µM) treatment dose-dependently restored cAMP accumulation, demonstrating comparable efficacy to the reference APLNR antagonist F13A (10^− 4^−100 µM) (Fig. 2D**)**. Moreover, Complementary calcium flux assays using the FLIPR^®^ system revealed that both candesartan and F13A competitively inhibited Apelin-13-mediated calcium mobilization, and quantitative analysis yielded IC_50_ values for both compounds (Candesartan: 1.214 µM; F13A: 1.174 µM) (Fig. 2E-F), indicating significant APLNR antagonistic activity.

To further analyze the binding capacity of candesartan to APLNR, we conducted surface plasmon resonance (SPR) analysis to determine the binding affinity between candesartan and APLNR. As shown in Fig. 2G, the response units (RU) increased significantly as the concentration of candesartan was raised from 0.390625 µM to 12.5 µM. This gradual increase in RU values indicates specific binding of candesartan to APLNR. The equilibrium dissociation constant (KD) calculated from the SPR data was 1.715 µM. Collectively, these results provide compelling evidence that candesartan is a potent APLNR inhibitor, capable of modulating cAMP signaling and binding specifically to the APLNR active pocket.

Candesartan Inhibits Angiogenesis by Suppressing Migration and Vascular Tube Formation of HUVEC

### Candesartan inhibits angiogenesis by suppressing migration and vascular tube formation of HUVEC

**Fig. 3:**
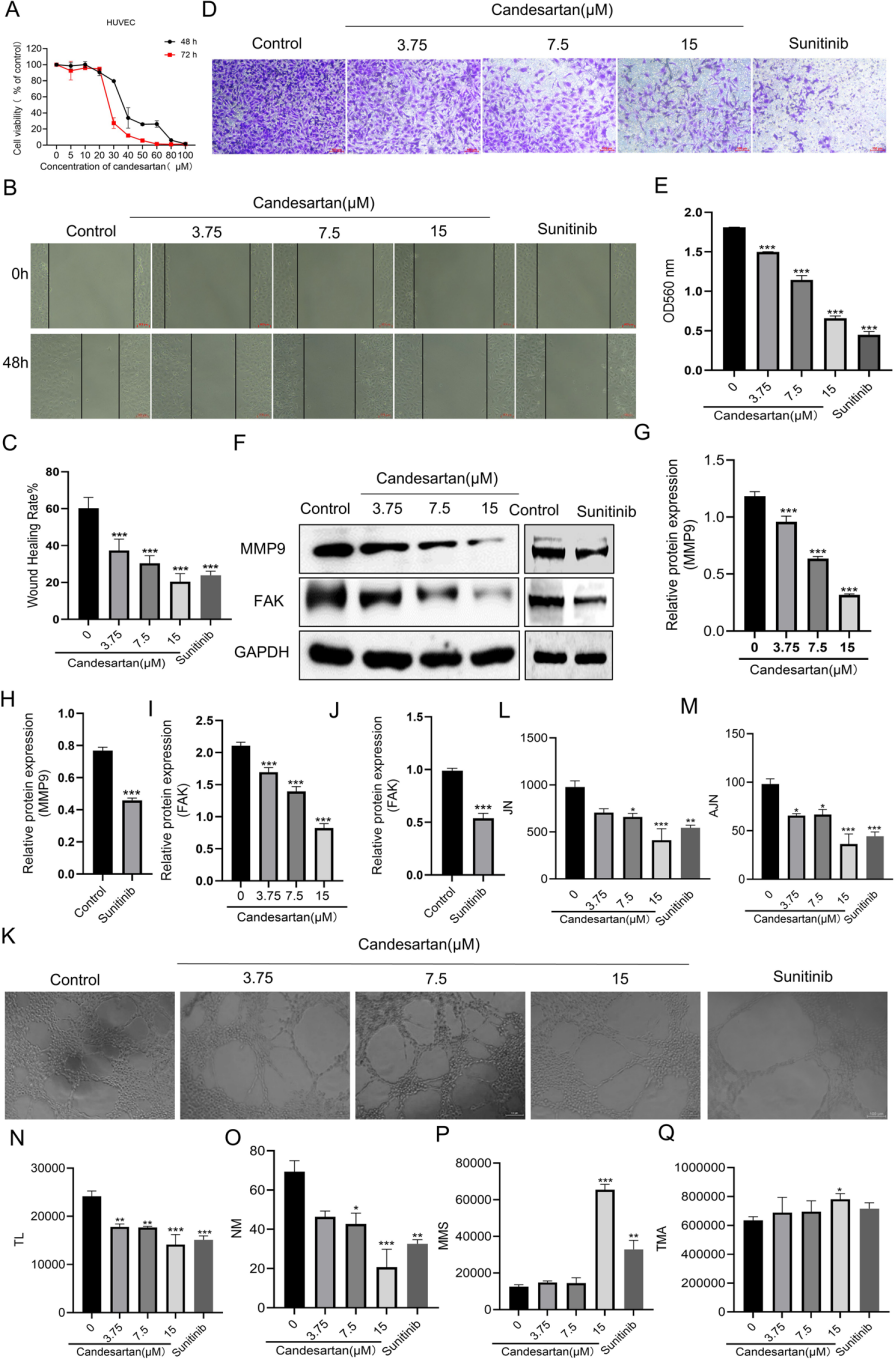
Candesartan inhibits angiogenesis and promotes vascular normalization in vitro. **A** The effect of Candesartan on the proliferation of HUVEC (*n* = 3). **B** and **C** wound healing assay was used to investigate the effect of candesartan (15, 7.5, and 3.75 µM) and sunitinib (2 µM) on the migration ability of HUVEC, scale: 100 μm. **D** and **E** Transwell assay was used to investigate the effect of candesartan (15, 7.5, and 3.75 µM) and sunitinib (2 µM) on the invasion ability of HUVEC, scale: 100 μm; **F-J** Western blotting was used to investigate the effect of candesartan (15, 7.5, and 3.75 µM) and sunitinib (2 µM) on the expression of FAK and MMP-9 in HUVEC. **K-Q** Vascular tube formation assay showed that both candesartan (15, 7.5, and 3.75 µM) and sunitinib (2 µM) can inhibit the angiogenesis and promote vascular normalization in vitro (JN: Number of Junctions, AJN: Number of Anchorage Junction, TL: Total Length, NM: Number of Meshes, MMS: Mean Mesh Size, TMA: Total Mesh Area). Each bar represents the mean ± SEM of three independent experiments. ^*^p < 0.05, ^**^p < 0.01, and ^***^p < 0.001 vs. the control group

The anti-proliferative effects of candesartan on human umbilical vein endothelial cells (HUVECs) were evaluated using the sulforhodamine B (SRB) assay. HUVECs were treated with graded concentrations of candesartan for 48 and 72 h, yielding IC_50_ values of 32.4 **±** 1.69 µM and 27.12 **±** 0.91 µM, respectively (Fig. 3A). Notably, candesartan at 15 µM exhibited no significant cytotoxicity on HUVEC cells, then the non-cytotoxic doses (15, 7.5, and 3.75 µM) were subsequently employed for further vascular normalization studies. Angiogenesis is a multi-step process requiring coordinated endothelial functions, such as cell migration, proliferation, and extracellular matrix remodeling [19]. In the experimental design, sunitinib, a VEGF antagonist, was included as a positive control. Then, at first, wound healing and transwell assays were performed to assess the inhibitory effect of candesartan on HUVEC cell migration. The results indicated that both candesartan and sunitinib significantly reduced the migratory and invasive capacities of HUVECs, with candesartan exerting a pronounced dose-dependent inhibitory effect (Fig. 3B-E). Additionally, the activation of FAK-MMP9 signaling is significantly correlated with the migratory and invasive potential of endothelial cells and is further involved in tumor metastasis [20]. And the western blot experiments revealed that both candesartan and sunitinib significantly decreased the expression of MMP9 and FAK in HUVEC (Fig. 3F-J). Moreover, in the later stages of angiogenesis, vascular endothelial cells undergo proliferation and migration and further align to form cord-like, lumen-like structures(21). Thus, we conducted a vascular tube formation assay to evaluate the effect of candesartan on vascular normalization. As shown in Fig. 3K-O, both candesartan and sunitinib significantly reduced the number of junctions, total segment length, and the number of meshes. These results indicated that candesartan can inhibit the tube formation ability of endothelial cells in vitro. It is worth noting that both candesartan and sunitinib could increase the mean mesh and the total meshe area of the small tube mesh (Fig. 3K, P-Q). In conclusion, the above results demonstrated that candesartan inhibited endothelial cell migration, invasion and tube formation at non-cytotoxic doses, suggesting its ability to inhibit angiogenesis and promote vascular normalization.

### Candesartan promotes tumor vascular normalization in vivo

**Fig. 4:**
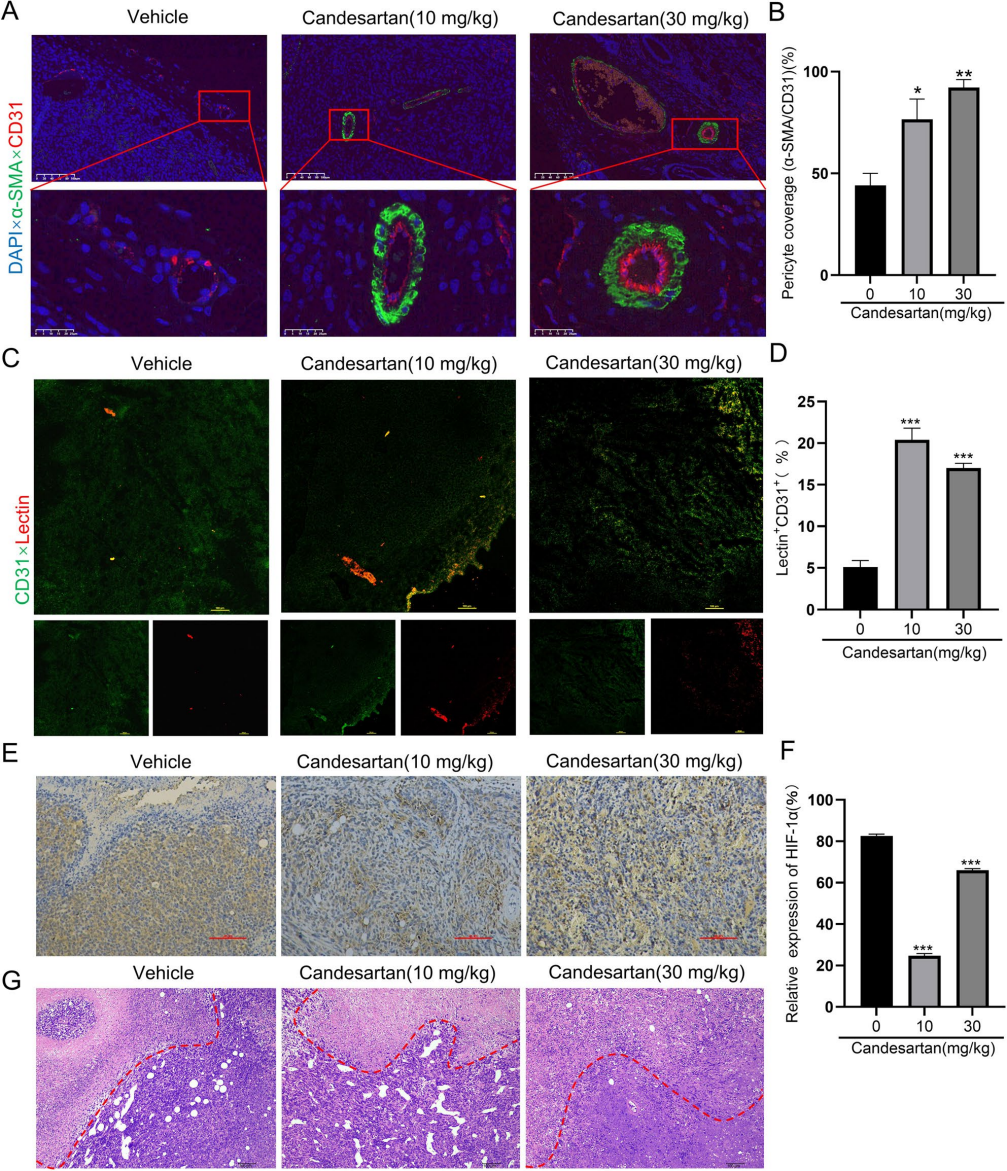
Evaluation of tumor vascular normalization in each treatment group and effects of candesartan on tumor vascular perfusion, tumor hypoxia, and tumor necrosis. **A** and **B** CD31 (red) and α- SMA (green) staining for endothelial cells and pericytes, respectively. Scale bars: 100 μm. **C** and **D** Tumor tissue sections were stained with Alexa Fluor^®^ 488-CD31 and DyLight™649 labeled tomato-lectin. (Green: CD31, red: tomotolectin, Scale bars: 100 μm); **E-G** Effect of candesartan on tumor hypoxia and necrosis, Scale bars: (E:500µM, G:100 μm). Each bar represents the mean ± SEM of three independent experiments. ^*^p < 0.1, ^**^p < 0.01, and ^***^P < 0.001, vs. the vehicle group

Based on the in vitro findings, we further established a breast cancer mouse model to evaluate the potential of candesartan in modulating Vascular Normalization in tumor tissues, Tumor neovasculature is pathologically distinct from normal vasculature, exhibiting characteristic immaturity that manifests primarily as deficient pericyte coverage [22]. To quantitatively assess vascular maturation, we performed dual immunofluorescence staining for the endothelial cell marker CD31 and pericyte marker α-SMA in tumor tissues. Quantitative image analysis revealed a significant increase in the coverage of α-SMA^+^ pericyte on CD31^+^ endothelial cell in candesartan-treated groups (10 mg/kg and 30 mg/kg, i.p.) compared to vehicle controls (Fig. 4A-B), indicating substantial improvement in vascular wall integrity and maturation. In addition, vascular perfusion indicates their transmission function is crucial for the delivery of chemotherapeutic and immunosuppressive agents [23]. To evaluate the effect of candesartan on tumor vascular perfusion function, DyLight™649-labeled tomato lectin (40 mg/kg), which specifically labels perfused blood vessels, was injected into the mice via tail vein injection 1.5 h before sacrifice. Then, perform frozen sectioning of the tumor tissue, microvessels were labeled with CD31 by immunofluorescence, and the co-expression percentage of tomato lectin and CD31^+^ was observed and calculated. As shown in Fig. 4C-D, only a small fraction of CD31^+^ blood vessels in the control group were coated with tomato lectin, with a low percentage of CD31^+^Lectin^+^. In contrast, the percentage of CD31^+^Lectin^+^ was significantly increased in candesartan-treated groups (10 mg/kg and 30 mg/kg), suggesting improved tumor vascular perfusion function.

Moreover, hypoxia is a common feature of the tumor microenvironment, with hypoxia-inducible factors (HIFs), particularly HIF-1α, playing a key role in the adaptive response to low oxygen levels, promoting angiogenesis and metastasis [24]. Results in Fig. 4E-G showed a significant decline of HIF-1α expression in tumor tissues from the candesartan group. Additionally, hematoxylin and eosin (H&E) staining of tumor sections revealed reduced necrotic areas in tumors treated with low-dose candesartan (10 mg/kg), while the high-dose (30 mg/kg) group showed a tendency towards increased necrosis, which was possibly due to excessive vessel pruning and exacerbated tumor hypoxia. Collectively, candesartan not only promotes the normalization of vascular structure and improves tumor vascular function but also alleviates tumor hypoxia. The significant therapeutic effects were observed in the low-dose group, demonstrating that candesartan can induce tumor vascular normalization in vivo.

### Candesartan activates the ROS/dsDNA/cGAS/STING pathway to promote vascular normalization

**Fig. 5:**
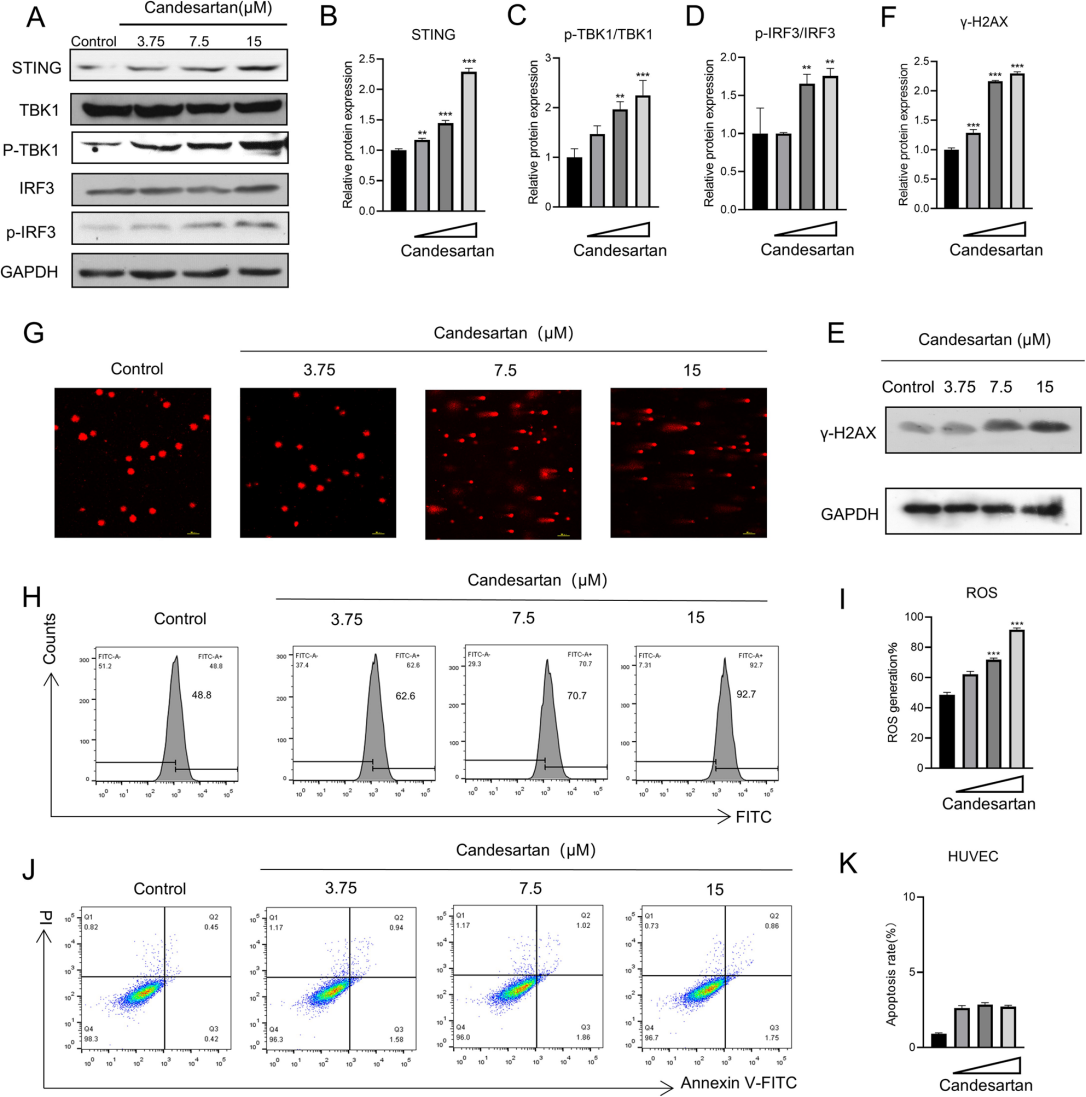
Candesartan promotes the normalization of tumor blood vessels by activating the STING pathway. HUVEC cells were treated with candesartan at different concentrations for 48 h, **A-D** Western blotting was used to detect the expression of STING pathway-related proteins. **E-F** Western blotting was used to detect the protein production of DNA damage marker γ-H2AX. **G** The effect of candesartan on DNA fragmentation in HUVEC cells was detected by comet assay. **H-I** Flow cytometry was used to analyze the effect of candesartan on ROS production in HUVEC cells. **J-K** The effect of candesartan on HUVEC apoptosis was detected by flow cytometry. Each bar represents the mean ± SEM of three independent experiments. ^**^p < 0.01, ^***^p < 0.001 vs. the control group

Previous studies have established that STING signaling pathway play a key role in modulating vasculature remodeling and tumor vascular normalization [25–27]. The mechanism involves the upregulation of both interferon-related genes and vascular stability genes, which has been demonstrated to improve vascular normalization in tumor tissues. Building upon this evidence, we investigated whether candesartan modulated vascular normalization through activating cGAS/STING signaling pathway. Firstly, we investigated the expression of the key proteins in STING signaling pathway. As demonstrated in Fig. 5A-D, candesartan treatment HUVECs for 48 h could induce dose-dependent upregulation of STING and its downstream signaling components, including the phosphorylation ratios of TBK1 (p-TBK1/TBK1) and IRF3 (p-IRF3/IRF3). These observations indicated that candesartan activated the STING signaling pathway. In addition, the activation of STING pathway is typically associated with the accumulation of cytosolic double-stranded DNA (dsDNA). Upon dsDNA detection by cyclic GMP-AMP synthase (cGAS), the cGAS-STING signaling cascade is initiated [28]. Given the observed results above, we further examined whether candesartan activates STING pathway by inducing DNA damage response. Western blot analysis revealed a dose-dependent increase in γ-H2AX expression **(**Fig. 5E-F**)**, a well-established biomarker of DNA double-strand breaks (DSBs). Consistent with these findings, comet assay results confirmed increased DNA fragmentation following candesartan exposure **(**Fig. 5G**)**. Moreover, Reactive oxygen species (ROS) are natural byproducts of cellular metabolism. Due to their highly reactive nature, ROS can damage DNA, proteins, and lipids. Additionally, Existing research has confirmed that, APLN-APLNR axis increases ROS generation in endothelia, vascular smooth muscle cell and human biliary cells [29–31].Thus, the effect of candesartan on ROS production in HUVEC cells was detected by flow cytometry. As shown in the Fig. 5H-I, ROS levels were gradually increased with increasing drug concentration. Given that high levels of ROS can induce apoptosis under conditions of redox imbalance, we examined the effect of candesartan on HUVEC cell apoptosis using flow cytometry. Results showed that no apoptosis was observed in HUVEC cells after candesartan treatment **(**Fig. 5J-K**)**.

**Fig. 6:**
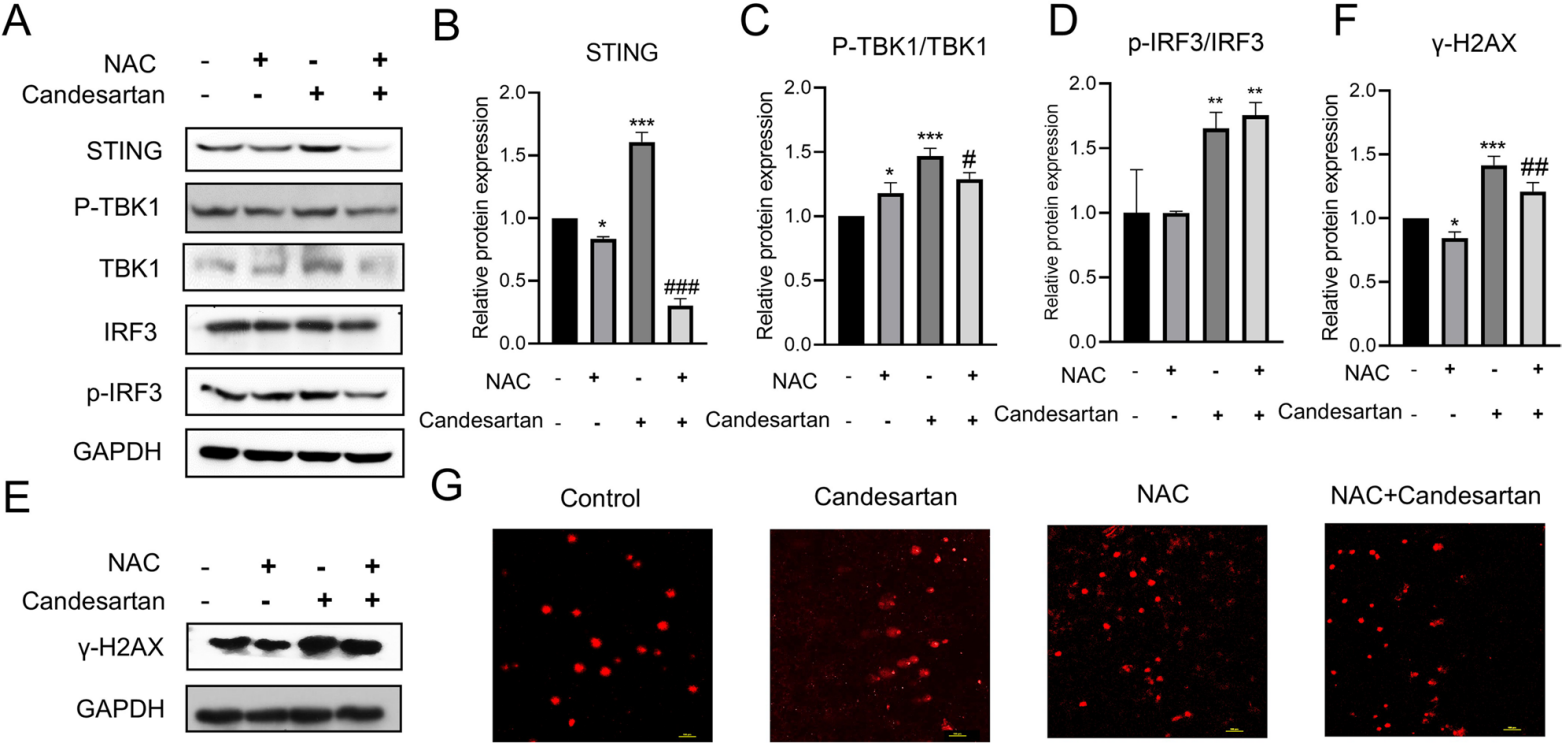
Effect of Candesartan on STING pathway after pretreatment with ROS inhibitor NAC. **A** HUVEC cells were treated with NAC (5mM) for two hours, then candesartan (15µM) was added for 48 h, and the expression of STING pathway-related proteins was detected by Western blotting; **B-D** Protein statistics in figure (A); **E** and **F** Western blotting detected the expression of γ-H2AX protein and the corresponding protein statistics; **G** After NAC pretreatment, the effect of Candesartan on DNA breakage of HUVEC cells was detected by comet assay. (*n* = 3, compared with the control group, ^*^P < 0.05, ^**^P < 0.01, ^***^P < 0.001; Compared with candesartan group, ^#^*P* < 0.05, ^##^*P* < 0.01, ^###^*P* < 0.001)

To further verify whether the increase of ROS causes intracellular DNA damage, which in turn activates the STING pathway, we conducted rescue experiments using the ROS scavenger N-acetylcysteine (NAC, 5 mM) in combination with candesartan (15 µM). Co-treatment of candesartan with NAC not only attenuated candesartan-induced upregulation of STING pathway components but also significantly reduced γ-H2AX expression (Fig. 6A-F). The comet assay results provided additional evidence that NAC pre-treatment could effectively mitigate candesartan-induced DNA damage (Fig. 6G). Collectively, these results demonstrate that candesartan induces DNA damage by increasing ROS levels in endothelial cells, which in turn causes double-stranded DNA breaks, promotes dsDNA accumulation, and activates the STING pathway to promote vascular normalization.

### Candesartan synergistically enhances the anti-tumor effect of sunitinib by inducing vascular normalization

**Fig. 7:**
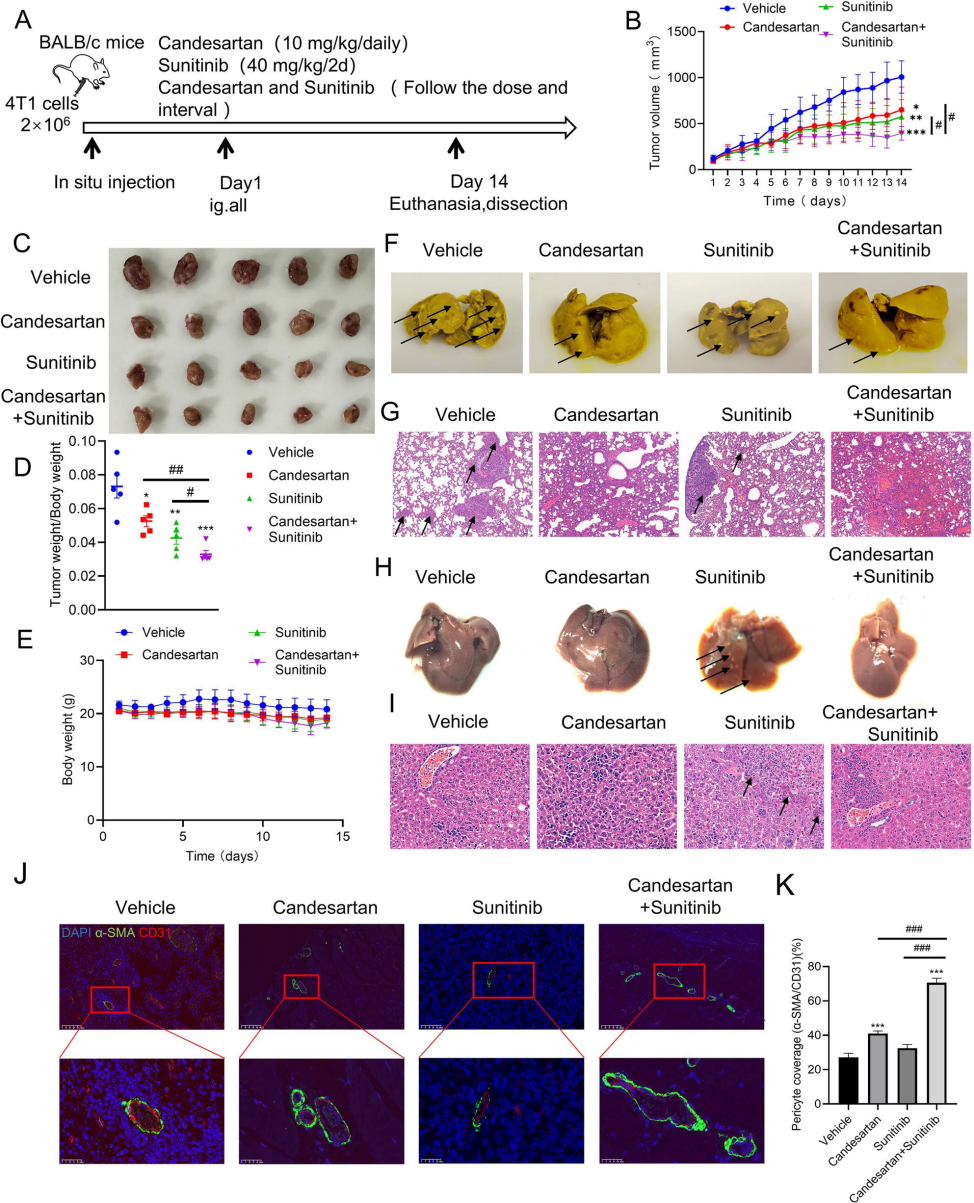
The anti-tumor efficacy of candesartan and Sunitinib in vivo. **A** Flow chart of animal experiments. **B** Statistical plot of tumor volume. **C** Tumor pictures of mice in each group. **D** The ratio of tumor weight to body weight in mice. **E **Statistical plot of body weight of mice in each groupn. **F** and **G** Candesartan alone or in combination with Sunitinib inhibited pulmonary metastasis of breast cancer (Scale bar: 100 μm). **H** and **I** Effect of candesartan combined with Sunitinib on mouse hepatic injury (Scale bar: 50 μm). **J** and **K** CD31 (red) and α- SMA (green) staining for endothelial cells and pericytes in tumors (Scale bars, 50 μm and 25 μm). Each bar represents the mean ± SEM, *n* = 5, compared with the vehicle group, ^*^P < 0.05, ^*^P < 0.01, ^***^P < 0.001 or compared with the candesartan + Sunitinib group, ^#^*P* < 0.05, ^##^*P* < 0.01, ^###^*P* < 0.001

Sunitinib, a VEGFR inhibitor, can inhibit angiogenesis. However, when used clinically for anti-tumor treatment, it may cause compensatory promotion of angiogenesis, resulting in abnormal and disordered tumor blood vessels, and vessel leakage, ultimately leading to adverse situation as tumor metastasis [32]. Given the previous findings that candesartan has the potential to induce vascular normalization. Therefore, we further explored whether combination with candesartan could enhance the anti-tumor efficacy of Sunitinib. Then, a breast cancer mouse model was employed to investigate the anti-tumor effects of candesartan and Sunitinib *in vivo.* The flow chart of animal experiments exhibited in Fig. 7A. BABL/c mice were randomly divided into four groups: vehicle, candesartan-treated (10 mg/kg), Sunitinib-treated (40 mg/kg), and the combination group. As shown in Fig. 7B-D, when compared with the vehicle group, both candesartan and Sunitinib could significantly reduce the tumor growth. Notably, the combination treatment group exhibited the most significant tumor growth inhibition. And each treatment group had no significant effect on the body weight of the mice (Fig. 7E). In addition, the combination treatment also significantly reduced the number of lung surface nodules, likely due to candesartan’s promotion of tumor blood vessel normalization and inhibition of breast cancer pulmonary metastasis (Fig. 7F-G).

Moreover, Sunitinib has been reported to transiently elevate liver enzymes, leading to fatal acute liver failure. Hepatotoxicity is a potentially fatal side effect of Sunitinib treatment, characterized by hepatocyte necrosis and inflammation [6]. In this study, we observed that the livers of mice treated with Sunitinib exhibited obvious hepatic injury (Fig. 7H-I). Histopathological analysis revealed that, after treatment with Sunitinib, hepatocytes showed patchy necrosis, pyknotic and fragmented nuclei, cytoplasmic dissolution, and extramedullary hematopoiesis was evident in local hepatic sinusoids. While, the treatment of candesartan combined with Sunitinib could significantly improve hepatic injury. These findings indicate that candesartan, when combined with Sunitinib, can mitigate Sunitinib-induced liver injury. This suggests that candesartan could serve as a potential adjuvant therapy to enhance the safety and efficacy of Sunitinib in cancer treatment.

Furthermore, we used the multi-immunofluorescence technique to examine the normalization of tumor blood vessels after drug treatment. As shown in Fig. 7J, results revealed that, in the candesartan-treated and combination group, the vascular structure becomes more normalized and the coverage of pericytes on endothelial cells significantly increased. Notably, the combination group showed a higher increase in pericyte coverage than the single-drug groups, indicating that the combination therapy could better promote the maturation and normalization of the tumor vasculature. In summary, the combination of candesartan with Sunitinib significantly enhanced anti-tumor efficacy and reduced pulmonary metastasis. This effect may be attributed to candesartan’s ability to promote tumor blood vessel normalization, thereby improving the therapeutic outcomes of Sunitinib. Importantly, candesartan mitigated Sunitinib-induced hepatotoxicity, suggesting its potential as an adjuvant therapy to improve both efficacy and safety in cancer treatment.

## Discussion

Vascular normalization has emerged as a promising strategy in anti-tumor therapy, addressing the abnormal characteristics of tumor blood vessels that often lead to poor drug delivery, hypoxia, and resistance to treatment. Induction of vascular normalization can enhance oxygenation, reduce interstitial fluid pressure, and improve the tumor delivery efficiency of anti-tumor agents. Currently, drugs that induce vascular normalization are mainly achieved through the selection of appropriate doses of anti-angiogenic drugs, such as drugs that target blocking of VEGFR. However, the clinical efficacy of these drugs is still not satisfactory [33]. Therefore, it is imperative to actively explore other drugs to induce vascular normalization. The APLN/APLNR system is involved in tumor angiogenesis, and APLNR antagonists have a significant effect on pathological neovascularization diseases. Our study contributes to this field by discovering novel APLNR antagonists and exploring the role of APLNR antagonists in modulating tumor vascular normalization and exploring its potential as an antitumor therapeutic agent.

Our study employed the drug repurposing strategies discovered that the existing approved drug candesartan act as a novel APLNR antagonist and exhibited significant potential in promoting tumor vascular normalization. Firstly, in a zebrafish retinal vascular abnormality model, where candesartan showed a trend towards normalizing the vasculature. In vitro experiments revealed that candesartan inhibited the proliferation, migration, and tube formation of HUVEC at non-cytotoxic doses. In addition, In vivo studies using a breast cancer mouse model further validated these findings. Candesartan treatment increased pericyte coverage on tumor vessels, improved vascular perfusion, and reduced tumor hypoxia. Mechanistically, candesartan activates the ROS/dsDNA/cGAS/STING signaling pathway, leading to increased pericyte coverage and improved vascular maturation. This pathway activation is crucial for the normalization process. Importantly, candesartan’s could enhance the anti-tumor effect of Sunitinib and mitigated Sunitinib-induced hepatotoxicity. This finding underscores the potential of candesartan as an adjuvant therapy to enhance the efficacy and safety of Sunitinib. The combination of candesartan with Sunitinib may provide a novel and effective treatment strategy, with the potential to improve both efficacy and safety. However, further research is needed to fully explore and validate these promising preliminary findings.

Furthermore, this study provided a comprehensive evaluation of candesartan’s effects on both in vitro and in vivo models, highlighting its potential as a novel anti-tumor agent by targeting APLNR. Candesartan is an angiotensin II receptor blocker, clinically used for the treatment of primary hypertension and with a well-documented safety profile in clinical use over 22 years. While, our study suggests that it may hold promise for repurposing for cancer therapy, particularly for cancer patients at risk of hypertension, candesartan could be a promising therapeutic drug. It is reported that Sunitinib Therapy may cause Treatment-Related Hypertension [3]. The ability of candesartan to normalize tumor vasculature while maintaining its antihypertensive properties positions it as a potentially valuable candidate for combination therapy with Sunitinib, though additional research is warranted to fully explore this potential.

## Conclusion

Our study identifies the clinically available drug candesartan as a potent inducer of tumor vascular normalization and demonstrates its synergistic efficacy with Sunitinib. The favorable safety profile of candesartan suggests that its combination with Sunitinib might be clinically manageable. While potential overlapping or novel toxicities need to be formally assessed in future clinical studies, extensive clinical experience on candesartan provides a strong foundation for designing such trials and managing anticipated adverse events. Our study indicated a new direction for the development of novel APLNR antagonists and also provided an effective strategy for cancer therapy by combining APLNR antagonists with Sunitinib or similar antiangiogenic agents. However, it is important to acknowledge the limitations of our study. Specifically, our findings are based on preclinical models, and the efficacy and safety of the candesartan-Sunitinib combination have not yet been evaluated in clinical trials. Future clinical studies are needed to fully validate these findings and to explore the full potential of this combination therapy. Additionally, the long-term effects and potential side effects of this combination in a diverse patient population remain to be determined. Therefore, while our results are promising, they should be interpreted with caution until confirmed in clinical settings.

## Supplementary Information

Supplementary Material 1

## Data Availability

All cell lines, vectors and other stable reagents generated in this study are available from the corresponding author. The datasets used or analyzed are available on reasonable request.

## Abbreviations

HIF-1: Hypoxia-inducible factor 1
FGF: Fibroblast growth factor
VEGF: Vascular endothelial growth factor
AAT: Anti-angiogenesis therapy
PASMCs: Pulmonary artery smooth muscle cells
sdAb: Single-domain antibody
GPCR: G protein-coupled receptor
SPR: Surface Plasmon Resonance
TCA: Trichloroacetic acid
SRB: Sulforhodamine B
NMA: Normal melting point agarose
LMA: Low melting point agarose
ROS: Reactive oxygen species
H&E: Hematoxylin and eosin
LD_50_: Lethal dose 50
Dpf: Days post-fertilization
RU: Response units
HUVECs: Human umbilical vein endothelial cells
p-TBK1/TBK1: Phosphorylation ratios of TBK1
p-IRF3: Phosphorylation ratios of IRF3
dsDNA: Double-stranded DNA
DSBs: DNA double-strand breaks
NAC: N-acetylcysteine
