# Supplementary material for: Novel APLNR antagonist candesartan induces tumor vascular normalization through ROS/cGAS/STING axis and augmented sunitinib response in breast cancer
Source: J Exp Clin Cancer Res. 2025 Nov 28;45:3. doi: 10.1186/s13046-025-03584-4 (PMC12763877; doi:10.1186/s13046-025-03584-4)
Supplement: Supplementary file 1 — Supplementary Material 1 [file 13046_2025_3584_MOESM1_ESM.docx]

**Table S1. Screening of APLNR Antagonists**

| Compound | Docking score | Structure | Inhibition rate  (2 μM, %) |
| --- | --- | --- | --- |
| A-1 | -9.8548 |  | 42.2 |
| A-2 | -9.03346 |  | 72.3 |
| A-3 | -10.2743 |  | 51.4 |
| A-4 | -9.02476 |  | 54.2 |
| A-5 | -11.6457 |  | 38.5 |
| A-6 | -10.7738 |  | 61.7 |
| A-7 | -8.64961 |  | 49.5 |
| A-8 | -10.7567 |  | 42.6 |

Table 1 (*Continued*)

| Compound | Docking score | Structure | Inhibition rate  (2 μM, %) |
| --- | --- | --- | --- |
| A-9 | -8.61279 |  | 65.4 |
| A-10 | -9.38452 |  | 37.6 |
| A-11 | -8.99035 |  | 48.1 |
| A-12 | -11.9305 |  | 37.4 |
| A-13 | -12.7644 |  | 29.8 |
| A-14 | -8.79091 |  | 47.6 |
| A-15 | -9.03533 |  | 51.1 |

Table 1 (*Continued*)

| Compound | Docking score | Structure | Inhibition rate  (2 μM, %) |
| --- | --- | --- | --- |
| A-16 | -10.6429 |  | 43.8 |
| A-17 | -9.92805 |  | 29.6 |
| A-18 | -10.5756 |  | 38.2 |
| A-19 | -10.9581 |  | 26.9 |
| A-20 | -9.26955 |  | 39.5 |
| A-21 | -14.7666 |  | 41.7 |
